# What Determines the Temporal Changes of Species Degree and Strength in an Oceanic Island Plant-Disperser Network?

**DOI:** 10.1371/journal.pone.0041385

**Published:** 2012-07-23

**Authors:** Aarón González-Castro, Suann Yang, Manuel Nogales, Tomás A. Carlo

**Affiliations:** 1 Island Ecology and Evolution Research Group, Consejo Superior de Investigaciones Científicas-Instituto de Productos Naturales y Agrobiología, La Laguna, Canary Islands, Spain; 2 Department of Biology, Pennsylvania State University, University Park, Pennsylvania, United States of America; University of Northampton, United Kingdom

## Abstract

Network models of frugivory and seed dispersal are usually static. To date, most studies on mutualistic networks assert that interaction properties such as species' degree (*k*) and strength (*s*) are strongly influenced by species abundances. We evaluated how species' degree and strength change as a function of temporal variation not only in species abundance, but also in species persistence (i.e., phenology length). In a two-year study, we collected community-wide data on seed dispersal by birds and examined the seasonal dynamics of the above-mentioned interaction properties. Our analyses revealed that species abundance is an important predictor for plant strength within a given sub-network. However, our analyses also reveal that species' degree can often be best explained by the length of fruiting phenology (for plants degree) or by the number of fruiting species (for dispersers degree), which are factors that can be decoupled from the relative abundance of the species participating in the network. Moreover, our results suggest that generalist dispersers (when total study period is considered) act as temporal generalists, with degree constrained by the number of plant species displaying fruits in each span. Along with species identity, our findings underscore the need for a temporal perspective, given that seasonality is an inherent property of many mutualistic networks.

## Introduction

Ecological communities are collections of interacting species that vary in space and time, and such species' relationships in a community can be modelled as networks where nodes are different species, and edges represent the interactions among them. The last decade witnessed an increase in the number of studies of plant-animal mutualistic networks, their properties, and their assembly mechanisms, e.g. [1]–[6]. Different parameters have been used to describe interactions among species in networks, such as species degree (*k*) [7] and species strength (*s*) [3]. The node (i.e., species) degree depicts the number of different species interacting directly with a particular species in the community. In a mutualistic network with two parties (e.g. birds and plants), the strength of a bird species quantifies the mutualistic services this species provides to the plant community (see Methods).

To date, two main hypotheses have emerged to explain observed patterns in such interaction networks: the ‘abundance’ and the ‘forbidden links’ hypotheses. The abundance hypothesis states that species within a community interact randomly. Therefore, the more abundant species will be sampled more frequently and, hence, will have a higher degree and strength than less abundant e.g. [1], [4]–[6], [8]. Moreover, it has been proved that fruit abundance could influence the local frugivorous bird abundance [9]–[11] and, hence also their interaction properties (*k* and *s*) [12]. On the other hand, the forbidden links hypothesis states that interaction patterns result from species-specific traits (phenological, morphological, physiological, etc.) which constrain the probability of interactions between potential mutualistic partners e.g. [2], . Nowadays it is accepted that the two hypotheses contribute in some extent to the observed patterns in mutualistic communities, both plant-pollinator [15] and plant-frugivore [12], [16] systems. However, few studies have sought to distinguish between the separate effects of abundance and the mere presence of species (e.g. fruiting phenology length) on species degree and strength, but see [17].

In addition, it has been demonstrated that seasonality is an inherent property in some plant-animal mutualistic communities because interactions are influenced by temporal changes of species abundance and/or species turnover [12], [17]–[23]. However, network models are usually static representations, and many unanswered questions remain regarding the relationship between temporal variability and network properties [24]. Despite the obvious and expected importance of seasonality, most studies that focus in temporal dynamics of mutualistic networks are related to plant-pollinator systems [17], [19]–[23]. In contrast, such an approach has received practically no attention in frugivory and seed dispersal networks, but [12].

A great number of studies focus on relatively large (complex) networks. Besides forbidden links (i.e. non observed links due to constraints) [14], large networks suffer the problems of having missing interactions, usually for the rare species that require more sampling effort to detect their mutualistic partners [6], [14]. However, in smaller networks, such as those in small oceanic islands, it is easier to sample and detect a higher proportion of the potential links between species [6], [25]. Therefore it is feasible the effect of abundance to be less striking in such smaller communities.

Here we report on a two-year study conducted in the thermophilous scrubland of the Canary Islands. We conducted an in-depth examination of the effects of several factors on two species interaction properties: species momentary degree (*mk* hereafter) and momentary strength, (*ms* hereafter) at different temporal sub-networks of plant-avian disperser interactions. Among factors explaining these interaction properties we focused on: the phenology length (i.e. the time during which fruits and avian dispersers are at the study site), the relative abundance of fruits and dispersers, the number (richness hereafter) of potential mutualistic partner species in the community and the species identity. Specifically, our goals are the following: **1)** To evaluate whether, at different temporal sub-networks, *mk* and *ms* are determined by the abundance of fruits and birds, or by the length of phenology, and/or the richness of potential mutualistic partners. **2)** To assess how the identity of species affects *mk* and *ms*. **3)** To determine if species that appear as generalists (i.e., high *k*) in an *overall* network (i.e., a network of interactions that is compiled over the entire years) are true generalists, or temporally specialized in sub-networks. Here we define ‘generalist’ as a species of bird or fruiting plant that interacts with many mutualistic partners [16].

Although degree and strength are usually correlated, we examine each parameter separately because mutualistic networks that are based on quantitative information are more heterogeneous than qualitative ones [16]. As degree is based on presence/absence of interaction, we expect species degree to be more strongly predicted by phenology length and/or richness of potential mutualistic partner species in the community (both based on the presence of species) than by abundance, which is based on fruits and dispersers density. Conversely, we expect strength (based on number of seeds dispersed) to be more strongly predicted by abundance. On the other hand, according to the forbidden link hypothesis [2], [13], [14], we also expect species identity to be a significant predictor of both degree and strength (Table 1).

**Table 1:** Explanatory variables used to predict the temporal variation of species interaction properties, and their associated hypothesis.

Response variable	Explanatory Variable	Associated Hypothesis
Plant interaction properties: *mk* and *ms*	Plant species identity	Every plant species could have different fruit traits that attract dispersers more or less intensively, therefore causing different interaction properties.
	Fruit relative abundance	Temporal variation in fruit abundance would lead to changes in plant interaction properties. The most abundant would have the highest *mk* and *ms*.
	Fruiting phenolgy length	Plants displaying fruits for longer periods could have a higher probability of interacting with different disperser species, increasing their *mk* and *ms*.
	Richness of disperser species	Increasing the number of disperser species in the habitat would produce a higher probability of seed dispersal interaction of plants and hence an increasing on their *mk* and *ms*, but especially on *mk*.
	Disperser abundance	Increasing disperser abundance would lead to increasing plant-disperser interaction probability, thus a higher *mk*, and especially *ms* are expected.
Animal interaction properties: *mk* and *ms*	Animal species identity	Animal species could have different behavioural, physiological and morfological adaptations toward frugivory, which would lead to different interaction properties.
	Disperser relative abundance	More abundant species should have a higher probability of interacting with plants, therefore higher *mk* and *ms*.
	Disperser phenology length	Dispersers persisting for a longer time in the habitat could have more time to interact with different plant species, increasing their *mk* and *ms*.
	Richness of fruiting plant species	Increasing the number of plant species in the habitat would produce a higher probability of seed dispersal interaction of dispersers and hence an increasing on their *mk* and *ms*, but especially on *mk*.
	Fruit abundance	Increasing fruit abundance would lead to increasing the plan-disperser interaction probability and hence to a higher *mk* and *ms* of dispersers.

*mk*: Plant/Animal momentary degree, *ms*: Plant/Animal momentary strength.

## Materials and Methods

### Study area

The study was carried out during two different years (Jun 2008–May 2009 and Jan 2010–Dec 2010) in a 4 ha patch of thermophilous shrubland, located at the northwest of the island of Tenerife (Canary Islands, UTM: 28R 317523 E/3138253 N, 220m a.s.l.). The climate is Mediterranean, with mean annual rainfall ranging between 200–400 mm and mean temperature between 16–19°C. The assemblage of species studied on this habitat is closely related with that present in the Mediterranean Basin. Fleshy-fruited plants community is composed of species such as *Asparagus plocamoides*, *Rubia fruticosa*, *Rhamnus crenulata* and *Pistacia atlantica*. In addition, the introduced species *Opuntia maxima* and *O. tomentosa* are present in the study site and their seeds appear in the plant-avian disperser network. The assemblage of native birds dispersing seeds is a subset of those inhabiting continental Mediterranean habitats (*Sylvia atricapilla*, *S. melanocephala*, *Turdus merula*, *Erithacus rubecula* and the occasional seed disperser *Cyanistes teneriffae*). However, they are mostly wintering and/or migrant in the continent, whereas in the Canary Islands they are year-round residents. Although the presence of migrant and wintering bird species, like *Turdus philomelos*, *T. torquatus*, *Phoenicurus phoenicurus*, *Sylvia cantillans*, etc. has been reported in the Canary Islands [26], there is no evidence of any significant presence of such dispersers in the thermophilous shrubland.

### Mutualistic interactions sampling and construction of temporal sub-networks

In our system, the seed dispersal network is more likely to represent the plant-disperser mutualism than frugivory networks, because several frugivorous bird species do not disperse seeds (e.g., pluck the fruit pulp without removing seeds or break seeds before rejecting them). In order to characterize the seed dispersal network, we focused on undamaged seeds in faeces, obtained from birds captured using mist nets placed in the study area every 15 days from dawn to dusk. The sampling effort was constant across the different temporal slices and the same for the two study years. To calculate the unit of effort, we multiplied mist-net length by the number of hours they were operative. Faecal samples were analysed with a dissecting scope for seed remains, which were counted and identified at species level, except for seeds of the genus *Opuntia*, which were considered as *Opuntia* sp.

For every temporal slice, we constructed quantitative networks (based on the number of dispersed seeds; Appendix S1), and calculated two species level interaction properties: species momentary degree (*mk*) and species momentary strength (*ms*). Due to the small size of the network, we focus on species-level properties, because using network-level measures are most appropriately applied to large and complex networks. A plant-animal mutualistic network can be depicted as an interaction matrix where plants are represented, for example, in rows and animals in columns. The dependence of a plant species *i* on an animal species *j* is the value of the cell *ij* in the interaction matrix divided by the total interactions of the row where the plant *i* is represented. Subsequently, the strength of an animal *j* is the sum of dependences of all plant species on this animal [3]. Although strength is also called ‘interaction strength’ by other authors [12], this term has been defined differently in another study [4]. To avoid confusion, we use ‘species strength’ instead [3].

Not all bird species were captured in every mist-netting session despite their presence at the study site. Thus, very short temporal slices can suffer from having low pattern resolution. To deal with this problem we choose a temporal resolution of three-month slices based on the average time that plant species display fruits in the studied habitat (Appendix S2) and according with each season of the year. Moreover, if we would consider smaller temporal slices, we would lose information regarding variation of phenology length, the explanatory variable we want to test against the abundance, which is one of the main goals of this study.

As species strength is based on frequency of interactions, it does not directly assess the impact of species on the demography of their mutualists. However, focusing on a seed dispersal network, rather than a frugivory network, means that we have a reasonable chance of estimating the demographic effect of dispersers on plants. Moreover, it has been demonstrated that interaction frequency is a good surrogate for the effect of mutualists on each other [27], and hence can be used to estimate the species strength in mutualistic networks [3], [27]. To calculate these interaction properties we used Bipartite 1.12 package [28], implemented in R 2.11 [29].

### Modelling temporal variation of interaction properties

We used a Generalized Linear Mixed Model (GLMM), with species identity as random effect variable, to model the variation of species properties (*mk* and *ms*) as a response to changes in our explanatory variables. To model *mk* we used the lme4 package [30] implemented in R, which allowed us to model the response variable (*mk*) with a Poisson error distribution. On the other hand, to model *ms* we used the nlme package [31] implemented in R, which allowed us to model the response variable with a Gaussian error distribution.

### Fruit abundance and phenology length

A given species can be abundant in two ways: producing many fruits or individuals in a given area (density), or having a long phenology, which in some extent is species-dependent. In this paper we will consider abundance as a quantitative measure (based on density) and phenology length as a qualitative one (presence/absence of a given species). To assess fruit abundance for every temporal slice we used 20 plots of 5 m^2^ randomly placed. We visited every plot monthly and estimated the number of fruits · m^−2^ for every plant species by visual counting method [18]. For each three month slice, we estimated the cumulative abundance and then calculated the relative fruit abundance for every plant species as the percentage of fruits of each species from the total community-wide fruit crop (Appendix S2). We also estimated seed abundance by multiplying the fruit abundance per the mean number of seeds per fruits. However, models performed better when fruit abundance instead of seed abundance was considered. Thus we use the fruit abundance in our models.

To evaluate the fruit persistence in the habitat, presence of fruits of each plant species was noted in a 500 m transect every 15 days. In this way, we obtained an approximate length of fruit display (in 15-days intervals). Therefore, this variable was categorized at different temporal length levels (15, 30, 45, 60, 75 and 90 days). Finally, we also noted the number of plant species displaying fruits in every temporal slice.

### Disperser abundance and phenology length

In order to relate bird abundance (individuals · m^−2^) with captured birds, we performed a simple regression analysis every 100 hours of sampling: Individuals·m^−2 = ^2.15+4.177 · (100 ·*C*), *P* = 0.001, *N* = 152, where *C* is the number of captured birds per unit of effort. To build this regression we used unpublished data from the same study area. The multiplication by 100 is to avoid very small decimal values of *C*. To take into account potential competition between disperser birds for fruits, our analyses used relative disperser abundance (likewise with fruits; Appendix S3).

Although birds are resident in the context of Canary Islands, they usually move across habitats, along altitudinal gradient, within the island. Therefore, bird persistence could vary across temporal slices. As mist-netting sessions were performed every 15 days, we also categorized the bird persistence for every three-month slice in the same way as fruit persistence. Because a lack of bird captures does not imply there were no individuals, we only classified absences as zeros if a species was not captured on three consecutive mist-netting sessions.

Birds studied in this work were caught using the mist-netting standard procedure approved by the “Centro de Migración de Aves (CMA)” of the “Sociedad Española de Ornitología” (SEO/BirdLife; personal license number 800032). Bird species studied in this work are listed in the UICN red list as “Least Concern”, thus extraordinary methods of management were not necessary. All necessary permits were obtained for the described field studies. The Cabildo (island council) of Tenerife (permission number 1203/2008), the Canary Islands Government (permission number 246699) and the landowner, Mr. Teobaldo Méndez, provided permission to work at the study site, which is located at the border of “Parque Rural de Teno”.

## Results

### Node momentary degree (mk)

**Figure 1:**
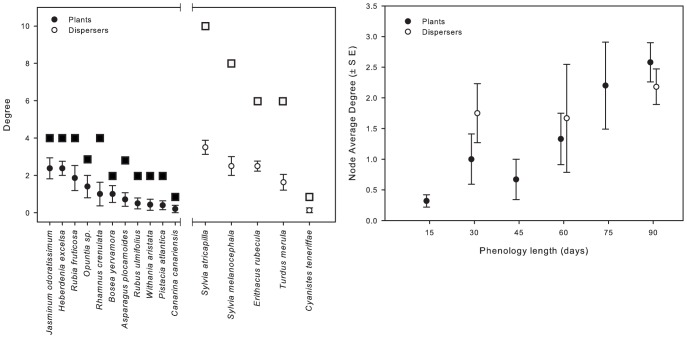
Plant and disperser degree. (a) Circles and error bars represent average momentary degree (*mk*) and standard error. Squares represent total degree (*k*) after two study years. Species increased its degree in accumulative manner, especially dispersers, whose degree was constrained by the number of plant species displaying fruits. Degree was variable among species, which demonstrates differences according with species identity. (b) Relationship between average *mk* (± SE) and phenology length. The *mk* increased with the phenology length, especially for plant species.

For all plant and disperser species, the average *mk* was lower than the total degree (*k*) after two years of study (Fig. 1a). However, for plant species, the average *mk* was closer to the total degree than for dispersers, which was clearly higher, except for the disperser *Cyanistes teneriffae*.

For plant species, their identity and fruiting phenology length were the two significant predictors of *mk* (Table 2). Among them, fruiting phenology length was the most important predictor of plant *mk* (Fig. 1b). However, abundance of both fruiting and avian species, and the richness of avian dispersers were not significant predictors of plant *mk* in the temporal sub networks (Table 2). For avian dispersers, species identity and the richness of fruiting plant species bearing fruits at each temporal slice significantly predicted *mk* (Table 2). As for plant species, abundance of both fruits and dispersers were not significant predictors of the *mk* of avian dispersers. Contrary to plant species, however, the phenology length of avian dispersers was not important for their *mk* in the temporal slices (Table 2 and Fig. 1b).

**Table 2:** Models with explanatory variables explaining species interaction properties: momentary degree (*mk*) and strength (*ms*) for both plants and dispersers.

Plant interaction properties			
Plant momentary degree	*LR*	*d.f.*	*P*-value
Plant species identity	20.76	10	0.023
Fruit relative abundance	0.196	1	0.66
Fruiting phenology length	30.54	1	<0.001
Total dispersers abundance	0.038	1	0.85
Richness of avian dispersers	0.276	1	0.59
**Plant momentary strength**			
Plant species identity	5.92	10	0.015
Fruit relative abundance	4.04	1	0.044
Fruiting phenology length	8.33	1	0.004
Total dispersers abundance	0.133	1	0.72
Richness of avian dispersers	0.313	1	0.58

Disperser interaction properties			
Disperser momentary degree	*LR*	*d.f.*	*P*-value
Disperser species identity	32.69	4	<0.001
Disperser relative abundance	1.43	1	0.23
Disperser phenology length	0.66	1	0.42
Total fruits abundance	0.45	1	0.5
Richness of fruiting species	4.03	1	0.043
**Disperser momentary strength**			
Disperser species identity	14.63	4	<0.001
Disperser relative abundance	0.41	1	0.52
Disperser phenology length	0.65	1	0.42
Total fruits abundance	0.28	1	0.59
Richness of fruiting species	0.68	1	0.41

Statistic of the Likelihood Ratio test (*LR*), degrees of freedom (*d.f*.) and significance level (*P*-value) for each explanatory variable in the model are shown.

### Node momentary strength (ms)

**Figure 2:**
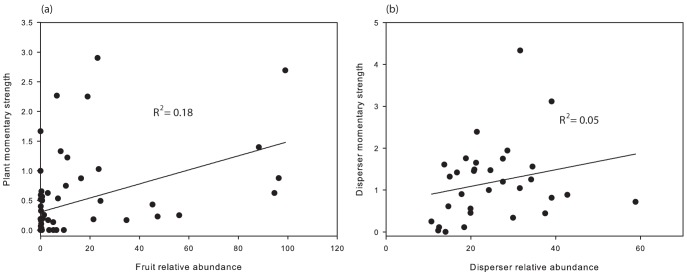
Relationship between momentary strength (*ms*) and relative abundance. The higher is the abundance of a given species at each temporal slice, the higher is its *ms* for both plants (a) and dispersers (b). However, this relationship was not very high; perhaps due to the stronger effect of other variables such as species identity or phenology length.

Variables explaining the *ms* of plants were plant species identity, relative fruit abundance, and the fruiting phenology length (Table 2). Neither avian disperser abundance nor the richness of avian species had significant effects on plant *ms* (Table 2). In the case of avian dispersers, the only explanatory variable significantly affecting their *ms* was the species identity (Table 2). Although we found a positive trend between avian disperser *ms* and their relative abundance at the temporal slices (Fig. 2b), this effect was not significant (Table 2).

## Discussion

Our analyses reveal that species interaction properties (i.e., *mk* and *ms*) for avian dispersers and fruiting plant species that participate in seed dispersal networks varied over time hand-in-hand with changes in their phenology length and in the richness of potential mutualistic partner species. This is a key finding because previous studies have suggested that the abundance of species is the most important factor explaining the interaction properties of species participating in mutualistic networks [1], [4]–[6], [8], [12], [32]. Our study shows that the length of the phenological pattern of fruiting plants species could be even more important than the abundance of species, calling attention to the importance of using network approaches that account for temporal variability in the analyses.

In our study system, bird degree (k in the overall network) can misrepresent the actual dispersal services provided at any temporal slice, because momentary degree (*mk*) is constrained by the richness of available fruiting plant species in the shorter time slices of the temporal sub-networks. Birds dispersed more plant species and with a higher frequency in the Spring and Summer sub-networks (Appendix S1), when more plant species displayed ripe fruit (Appendix S2). It is clear that the richness of partner species, not only the abundance can affect the species' degree in a frugivory and seed dispersal network, and it is curious why so little attention previous studies have given to ‘species richness’ as an explanatory variable, but see [33]. If dispersers' abundance is related to the number of plant species displaying fruits, but the last variable is not measured, this could result in an overestimated role of how abundance explains species degree. Thus, a measure of relative degree, as proposed by [33], could be more informative about the species generalization level.

Previous studies e.g. [34] suggest that the generalization level of species is scale-dependent, with generalization increasing as the temporal and/or spatial scale of sampling is increased. Thus, it is possible that a temporal specialist will appear as a generalist from a global network perspective, because it gradually increases its degree throughout the year (Yang and Carlo unpub. data). However, our results suggest that some avian dispersers that will be classified as generalists in the global network are also generalists in the temporal sub-networks. On the other hand, a true specialist would consume a fixed and small subset of fruiting species irrespective of the fruiting species richness at the temporal sub-networks.

Other factors, such as the availability of other food resources (e.g. invertebrates) could also have influenced the *mk* of avian dispersers, especially those that are specialist. For example, *Cyanistes teneriffae* disperses few fruiting species and have diets dominated by other food items (e.g. invertebrates). Still, the rest of the passerine birds we studied are highly frugivorous in the Canary Islands, as well as in other regions they inhabit (e.g. *Sylvia atricapilla* [8], [12], *S. melanocephala* or *Erithacus rubecula* [12]). Thus, we believe that the availability of non-fruit food resources had a negligible effect on the degree of most avian dispersers in this archipelago.

For plant species we observed that neither the density nor the species richness of the avian dispersers increased the *mk* of plants in the networks. This could be due to the inherently low variability in the species richness of avian dispersers at the study site (Appendix S3). However, this effect could be significant in localities where there is greater turnover in the richness of disperser species across seasons [12]. On the other hand, the length of the fruiting phenology at each temporal slice had a significant effect on both the plant *mk* and *ms*. This finding agrees with findings of other studies in plant-pollinator systems [17], and must be underscored because previous studies on seed dispersal networks have not distinguished between abundance and phenology length effects on species interaction properties (e.g. *k* and *s*), especially because not all species with extended fruiting periods are abundant.

In the thermophilous shrubland, a high proportion of plant species (e.g. *Asparagus plocamoides* or *Rhamnus crenulata*; Appendix S2) produce fruit crops of low relative abundance. Thus, many plant species in this habitat may rely on bearing fruits for extended periods as a mean to increase interactions with avian dispersers. However, this relationship between fruiting phenology length and degree seems to be very variable across plant species (Appendix S4). Plant species relying on either high fruit abundance or on high fruit persistence may represents two distinct life-history strategies [35] for achieving connectivity in networks of frugivory and seed dispersal.

Although fruiting phenology length at each temporal slice is generally an important explanatory variable for plant momentary degree, fruit abundance can be more important for some plant species. For example, *Heberdenia excelsa* produced fruits throughout the entire year (Appendix S2), thus its fruiting phenology length did not vary. Therefore, differences in seed dispersal interactions for *H. excelsa* were not dependent on the length of the fruiting seasons, but rather on its relative abundance. This species was dispersed by more bird species and more frequently (number of seeds dispersed) in autumn and winter when its fruit relative abundance is the highest (Appendices S1 and S2). This suggests that whereas fruit abundance could be important for some species when fruiting length is invariant, the best overall predictor for differences in plants' degree and strength is the length of the fruiting period, especially for plants with relatively small fruit crops.

It is interesting to note that abundance of species and their mutualistic partners had only weak effects on species interaction properties. The only significant effect was of fruit relative abundance on plant *ms* (Table 2 and Fig. 2a). This result supports the abundance hypothesis [4]–[6], [8], as avian dispersers closely tracked and responded to shifts in the availability of fruit resources as they do elsewhere [11], [12], [18]. However, for avian dispersers, their abundance did not explain their degree or strength in the network. Species abundance is thought to affect species' degree and strength, because for highly abundant species we are more likely to detect interactions than for rare species, e.g. [1], [4]–[6], [8], [12]. However, our results suggest that abundance may not be a prerequisite to interact with more plant species.

Additional reasons for finding no effects of disperser abundance on interaction properties could be due to the methods used to estimate species abundance [5], or to the small size of the studied community [6]. Whereas frugivore abundances have been measured by other studies as the number of visit to fruiting plants, e.g. [8], we estimated abundance based on a method that combines information from mist-net capture rates and census data (see methods). Therefore, in this study, we avoid the problem of dependence between data on interaction frequency and data on species abundance. Still, other studies that have used similar methods to ours have found strong effect of species abundances [12]. This leaves the size of the network as a factor that could explain our findings [6], noting that correlations between the asymmetry of species abundance and the asymmetry of species interactions was higher in continental frugivory networks (i.e., larger networks) than in island ones (i.e., smaller networks) [36]. The detection of interactions in small communities is less prone to sampling biases that are inherent to larger communities [6], and it is possible that abundance effects appear to be stronger on large-sized networks due to sampling effects because rarer species are not sampled as well as common ones [4]–[6].

### Species and interaction properties

In addition to variables related to the abundance of species or their potential mutualistic partners, species identity was always significant as predictor of *mk* and *ms* of both plants and animals. Indeed, species identity was the only significant explanatory variable for disperser *ms*. For plants, species differ in their attractiveness for dispersers [18], [37], which can be explained to some extent by differences in the nutrient content of fruit of many Mediterranean plants [38]. Birds can also show preferences for fruit based on seasonal changes in their nutritional requirements [38], which can lead to differences in *mk* and *ms* of plant species. For example, in summer, when water is a more valuable resource, one of the most connected plant species is *Jasminum odoratissimum* (with 87.8% pulp water, a degree of 3 in 2008 and 4 in 2010 and a strength of 1.82 in 2008 and 1.22), whereas *Pistacia atlantica* has only 5.1% pulp water and is one of the least consumed species (degree of 1 in 2008 and 0 in 2010 and a strength of 0.02 in 2008 and 0 in 2010). This suggests that further network studies considering chemical composition of fruit pulp should be undertaken.

In the case of birds, the importance of species identity is consistent with the fact that different species have different morphological and physiological adaptations toward frugivory [12], [39], [40]. A previous study [12] found the same effect of species identity in a larger fruit-birds interaction network; this suggests that the effect of species identity may be independent of network size. Thus, the most frugivorous species will have the highest degree and strength in each temporal sub-network, whereas the least frugivorous can be constrained by morphological or physiological traits. For example, as noted above, *Cyanistes teneriffae* is a small passerine bird that eats both fruits and insects. Although it is the most abundant bird and consumes fruit pulp, it disperses few seeds (lowest *k* and *s*). This could be due to this bird's small gape width. Still, several small-seeded plants like *Rubus ulmifolius* can occasionally be dispersed by *C. teneriffae* (Appendix S1). For avian dispersers, the species identity was the only variable explaining their *ms*, suggesting that any effects of abundance (i.e., density of individuals or fruits) on this interaction property are also species-specific [12]. This is important to clarify because previous studies have not specified if effects of abundance or phenology length apply to any species in mutualistic networks, e.g. [1], [4]–[6], but see [12].

Last, we want to point out that a temporal sub-network perspective can be useful to better understand the structure and evolution of interaction networks. For example, some authors [2], [16] have proposed that mutualistic networks do not follow laws of preferential attachment (i.e., that species degree influences the acquisition probabilities of new interactions [41]). Instead, they propose that species interactions are heavily constrained by phenotypical traits, such as morphological, phenological or accessibility restrictions that create “forbidden links” in the network [2], [13], [14], [16]. An alternative hypothesis is that abundance by itself could provide an interaction rule [5]. Our results in the thermophilous scrublands of the Canary Islands support the forbidden link hypothesis, because probabilities of observing new species interactions depended more strongly on phenology length and other species-specific traits, rather than on the abundance of fruits and avian dispersers.

## Supporting Information

Appendix S1 **Temporal seed dispersal sub-networks.**
(PDF)

Appendix S2 **Fruit phenology during the two years at the study site.**
(PDF)

Appendix S3 **Temporal variation of bird abundance during the two study years.**
(PDF)

Appendix S4 **Plant momentary degree against fruiting phenology length.**
(PDF)
