# Supplementary material for: What Determines the Temporal Changes of Species Degree and Strength in an Oceanic Island Plant-Disperser Network?
Source: PLoS One. 2012 Jul 23;7(7):e41385. doi: 10.1371/journal.pone.0041385 (PMC3402460; doi:10.1371/journal.pone.0041385)

Appendix S1: Different temporal sub-networks at Los Adernos (Tenerife), where circles are species and links represent interactions among them. Left party represents plant species and right party animal species. Circles size is proportional to species abundance and links width to number of seeds dispersed. Only species participating at each temporal sub-network are represented. Abbreviation of names: ASPLO: *Asparagus plocamoides*, BOYER: *Bosea yervamora*, CANCAN: *Canarina canariensis*, HEBEX: *Heberdenia excelsa*, JASOD: *Jasminum odoratisimum*, OPUN: *Opuntia* sp., PISAT: *Pistacia atlantica*: RHACRE: *Rhamnus crenulata*, RUFRU: *Rubia fruticosa*, RUBUL: *Rubus ulmifolius*, WITAR: *Withania aristata*, SYLATRI: *Sylvia atricapilla*, SYLMEL: *Sylvia melanocephala*, ERIRUB: *Erithacus rubecula*, TURME: *Turdus merula*, CYATEN: *Cyanistes teneriffae*.

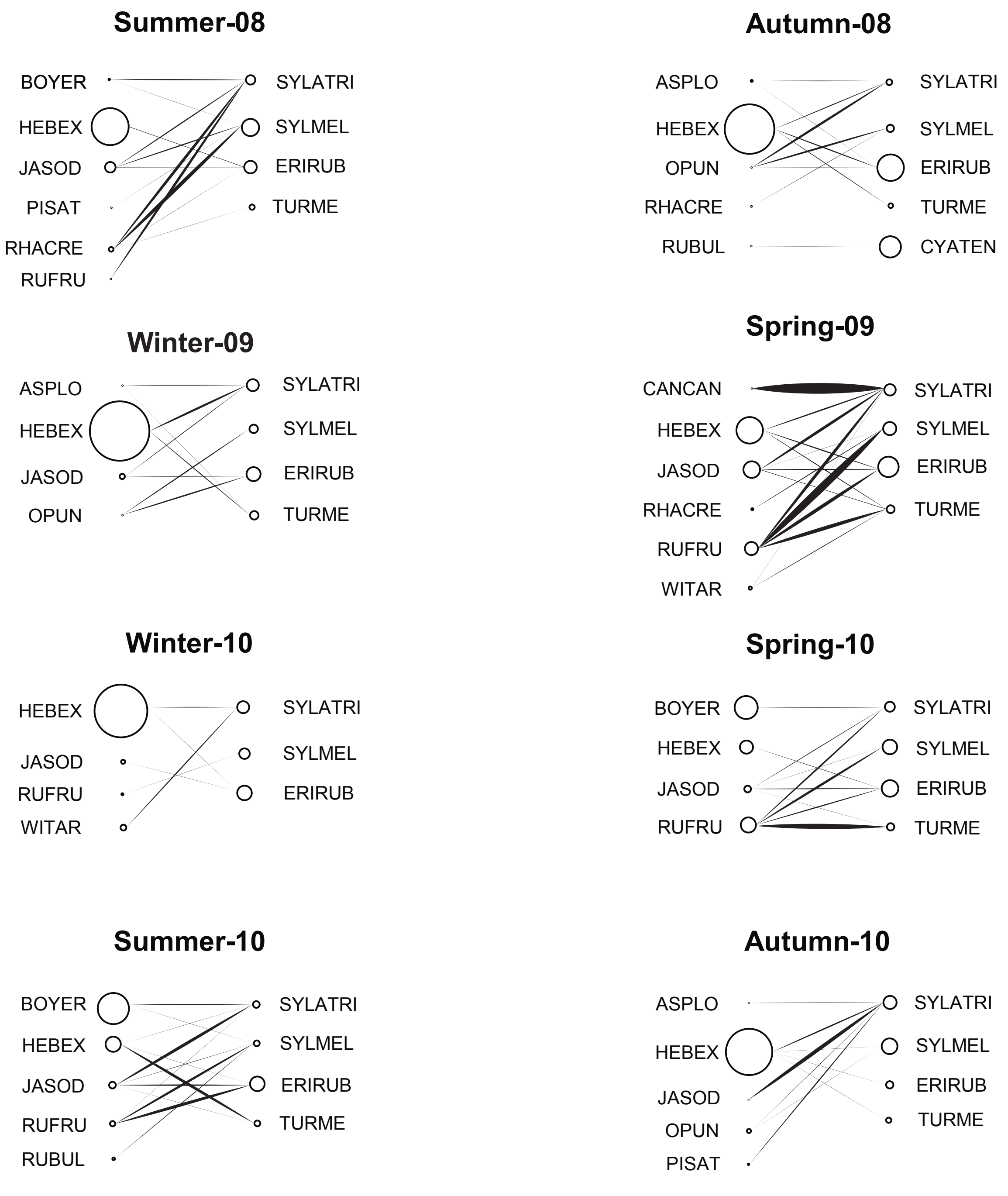

Supplement: Appendix S1 — Temporal seed dispersal sub-networks. (PDF) [file pone.0041385.s001.pdf]
