# Supplementary material for: What Determines the Temporal Changes of Species Degree and Strength in an Oceanic Island Plant-Disperser Network?
Source: PLoS One. 2012 Jul 23;7(7):e41385. doi: 10.1371/journal.pone.0041385 (PMC3402460; doi:10.1371/journal.pone.0041385)

Appendix S2. **Fruit phenology through the two years at the study site**. Left graphs show fruit abundance in 2008-2009. Right graphs show fruit abundance in 2010. a) plant species whose crop density reached more than 15 fruits/m^2 at least once during study periods. b) species whose production never reached 15 fruits/m^2.

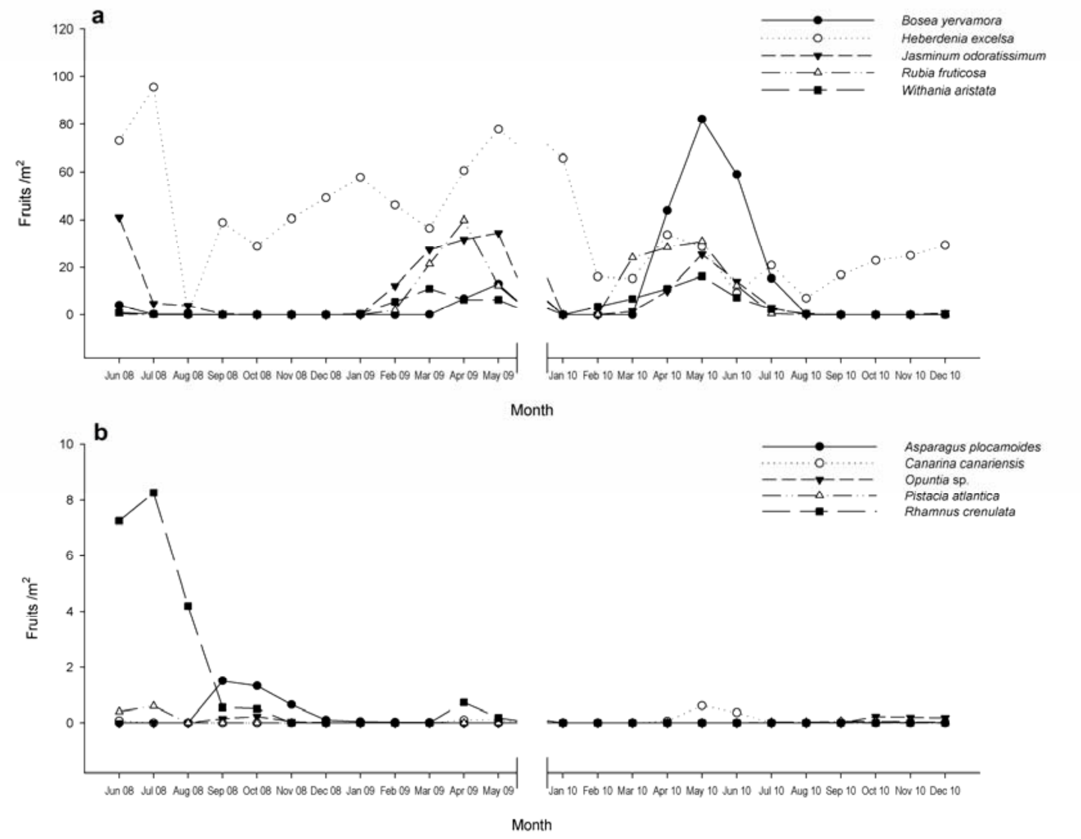

Supplement: Appendix S2 — Fruit phenology during the two years at the study site. (PDF) [file pone.0041385.s002.pdf]
