# Supplementary material for: What Determines the Temporal Changes of Species Degree and Strength in an Oceanic Island Plant-Disperser Network?
Source: PLoS One. 2012 Jul 23;7(7):e41385. doi: 10.1371/journal.pone.0041385 (PMC3402460; doi:10.1371/journal.pone.0041385)

Appendix S3. **Temporal variation of bird abundance during the two study years**. These graphs represent the temporal variation of disperser birds abundance throughout the two study years.

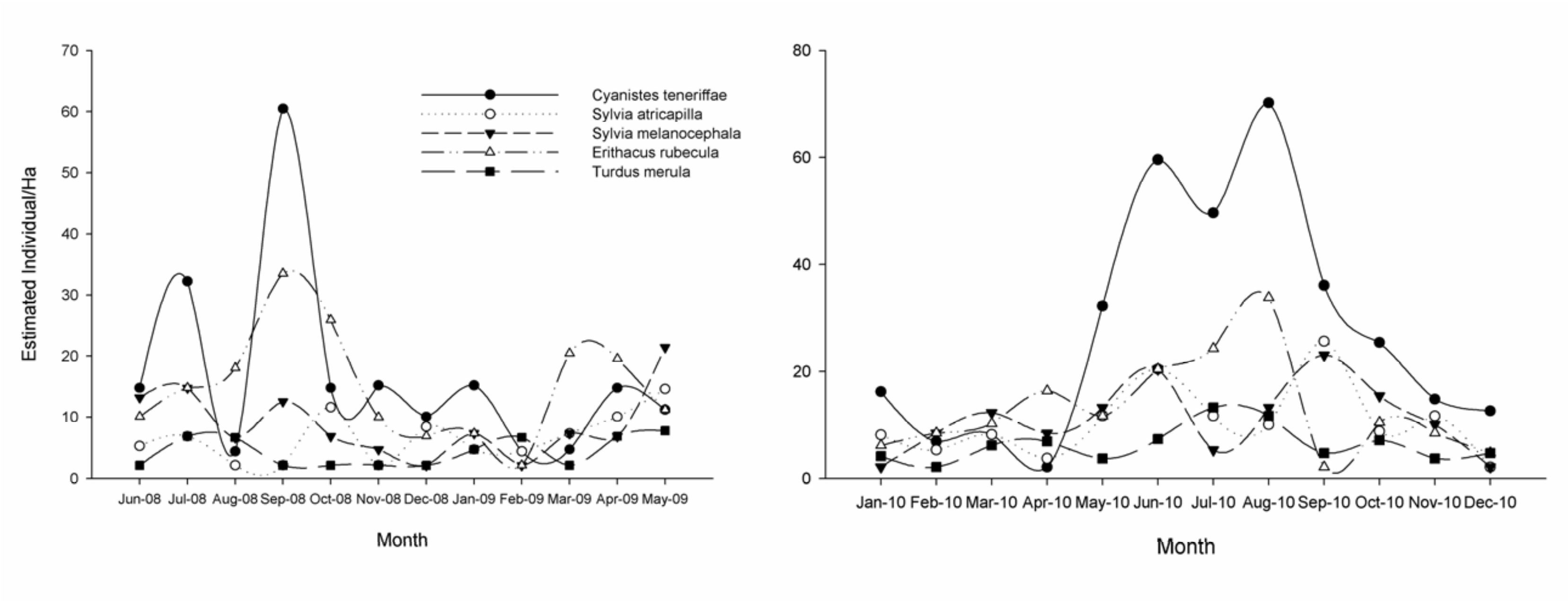

Supplement: Appendix S3 — Temporal variation of bird abundance during the two study years. (PDF) [file pone.0041385.s003.pdf]
