# Supplementary material for: What Determines the Temporal Changes of Species Degree and Strength in an Oceanic Island Plant-Disperser Network?
Source: PLoS One. 2012 Jul 23;7(7):e41385. doi: 10.1371/journal.pone.0041385 (PMC3402460; doi:10.1371/journal.pone.0041385)

Appendix S4. Plant momentary degree against fruiting phenology length. These graphs show the relationship between fruiting phenology length and degree at different temporal sub-networks for different plant species. For illustrative purposes we show only some cases that exemplify the different types of relationship between these two variables.

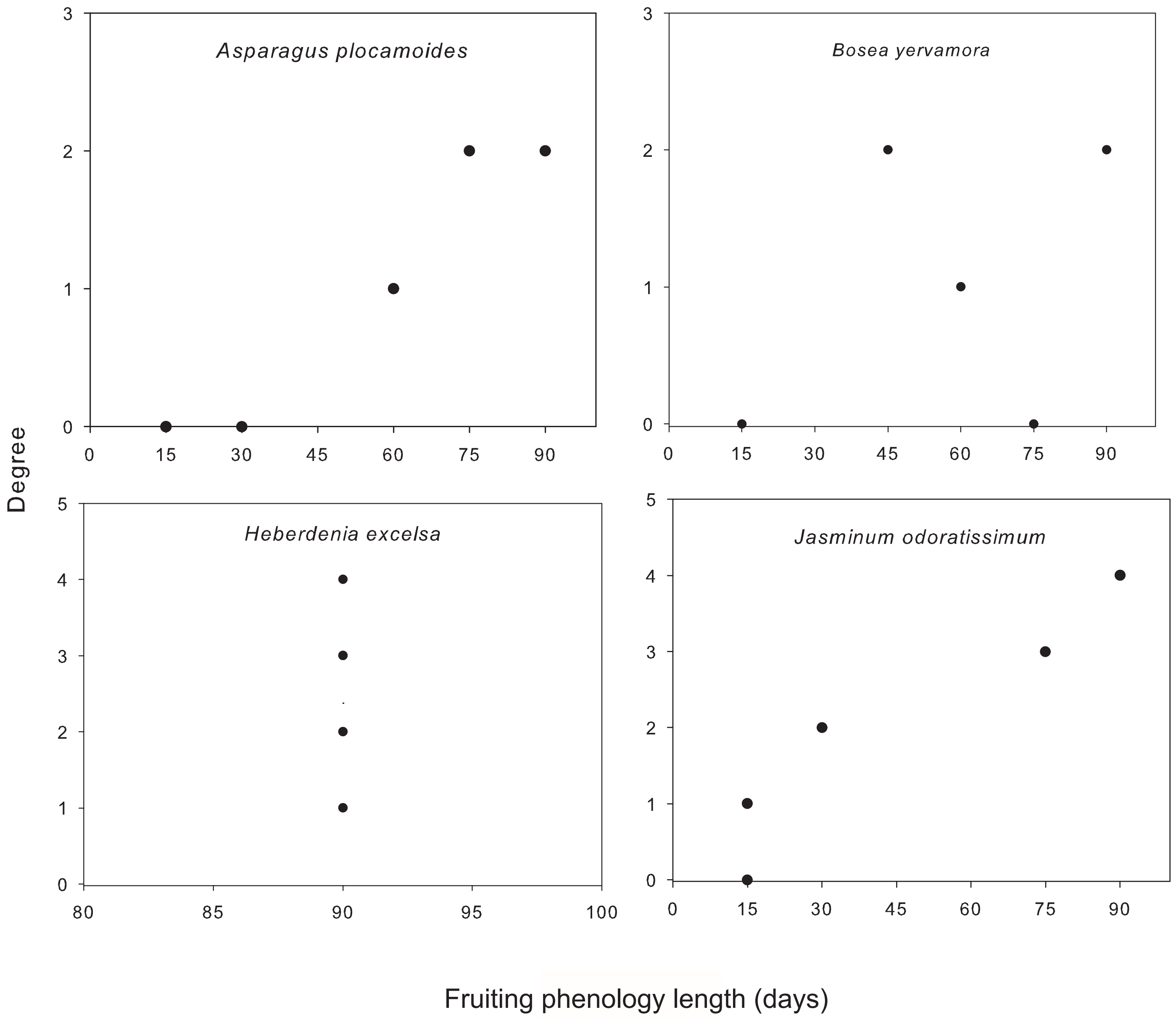

Supplement: Appendix S4 — Plant momentary degree against fruiting phenology length. (PDF) [file pone.0041385.s004.pdf]
